# ActEarly: a City Collaboratory approach to early promotion of good health and wellbeing

**DOI:** 10.12688/wellcomeopenres.15443.1

**Published:** 2019-10-14

**Authors:** John Wright, Andrew Hayward, Jane West, Kate Pickett, Rosie M. McEachan, Mark Mon-Williams, Nicola Christie, Laura Vaughan, Jess Sheringham, Muki Haklay, Laura Sheard, Josie Dickerson, Sally Barber, Neil Small, Richard Cookson, Philip Garnett, Tracey Bywater, Nicholas Pleace, Eric J. Brunner, Claire Cameron, Marcella Ucci, Steve Cummins, Daisy Fancourt, Jens Kandt, Paul Longley, Steve Morris, George Ploubidis, Robert Savage, Robert Aldridge, Dan Hopewell, Tiffany Yang, Dan Mason, Gillian Santorelli, Richard Romano, Maria Bryant, Liam Crosby, Trevor Sheldon

**Affiliations:** 1 Bradford Institute for Health Research, Bradford, BD9 6RJ, UK; 2 Institute of Epidemiology and Health Care, UCL, London, WC1E 6BT, UK; 3 Department of Health Sciences, University of York, UK, York, YO10 5DD, UK; 4 School of Psychology, University of Leeds, Leeds, LS2 9JT, UK; 5 Centre for Transport Studies, Department of Civil, Environmental and Geomatic Engineering, UCL, London, WC1E 6BT, UK; 6 Space Syntax Laboratory, Bartlett School of Architecture, UCL, London, WC1E 6BT, UK; 7 Extreme Citizen Science Group, Department of Geography, UCL, London, WC1E 6BT, UK; 8 University of Bradford, Bradford, BD7 1DP, UK; 9 Centre for Health Economics, University of York, York, YO10 5DD, UK; 10 York Cross-disciplinary Centre for Systems Analysis and School of Management, University of York, York, YO10 5GD, UK; 11 Centre for Housing Policy, University of York, UK, York, YO10 5DD, UK; 12 Department of Social Science, UCL Institute of Education, UCL, London, WC1H 0AA, UK; 13 UCL Institute for Environmental Design and Engineering, The Bartlett Faculty of the Built Environment, UCL, London, WC1H 0NN, UK; 14 Population Health Innovation Lab, Department of Public Health, Environments & Society, London School of Hygiene & Tropical Medicine, London, WC1H 9SH, UK; 15 Consumer Data Research Centre Department of Geography, UCL, London, WC1E 6BT, UK; 16 Primary Care Unit, Department of Public Health and Primary Care, University of Cambridge, Cambridge, CB1 8RN, UK; 17 Centre for Longitudinal Studies, UCL, London, WC1H 0NU, UK; 18 Institute of Education, UCL, London, WC1H 0A, UK; 19 Institute of Health Informatics, UCL, London, NW1 2DA, UK; 20 Bromley by Bow Centre, UCL, London, E3 3BT, UK; 21 Institute for Transport Studies, University of Leeds, Leeds, LS2 9JT, UK; 22 Leeds Institute of Clinical Trials Research, University of Leeds, Leeds, LS2 9JT, UK

**Keywords:** Environment and Public Health, Noncommunicable diseases, Child Health, Ethnicity, Mental Health

## Abstract

Economic, physical, built, cultural, learning, social and service environments have a profound effect on lifelong health. However, policy thinking about health research is dominated by the ‘biomedical model’ which promotes medicalisation and an emphasis on diagnosis and treatment at the expense of prevention. Prevention research has tended to focus on ‘downstream’ interventions that rely on individual behaviour change, frequently increasing inequalities. Preventive strategies often focus on isolated leverage points and are scattered across different settings. This paper describes a major new prevention research programme that aims to create City Collaboratory testbeds to support the identification, implementation and evaluation of upstream interventions within a whole system city setting. Prevention of physical and mental ill-health will come from the cumulative effect of multiple system-wide interventions. Rather than scatter these interventions across many settings and evaluate single outcomes, we will test their collective impact across multiple outcomes with the goal of achieving a tipping point for better health. Our focus is on early life (ActEarly) in recognition of childhood and adolescence being such critical periods for influencing lifelong health and wellbeing.

## Background

Areas with high levels of child poverty tend to have increased rates of obesity, polluted roads with low walkability, poor quality green spaces for play and exercise, high fast food outlet density and food poverty; poorer levels of child development, school readiness and educational attainment, higher school exclusion rates, poor performing schools and lower entry into further education; unsafe neighbourhoods, higher levels of youth crime, physical decay, poor public services and poor quality, overcrowded unfit, temporary/rented and unaffordable housing ^1, 2^. Children growing up in these areas are more exposed to stress, chaos, violence and household instability ^2^. These wider determinants and inequalities in these, damage child health and cause an accumulation of multiple environmental risks and clustering of unhealthy behaviours, that impair life opportunities and increase longer term non-communicable disease (NCD) risk ^1^. Addressing them can improve health outcomes ^3^, but too often NCDs have been attributed to bad choices rather than framed as emergent properties of complex systems. Public health interventions seek to directly influence behaviours rather than addressing the conditions that drive them and directly and indirectly affect health ^4^. There is robust research on how upstream factors affect NCD risk but little around how to address these at a local level where action is needed. There is also poor linkage between academics, those with broader interdisciplinary expertise, and the statutory, voluntary, cultural and commercial sectors, despite them all having a role in improving public health.

There is increasing focus on how to reduce inequalities in wider determinants ^5^, shifting the emphasis from deficits to harnessing of community assets, recognising the lived experiences and resourcefulness of disadvantaged communities, working ‘with’, rather than delivering ‘to’ people, and applying systems thinking principles to examine poor and unequal health “as outcomes of a multitude of inter-dependent elements within a connected whole” ^6, 7^. Components of factors influencing health, interact in complex and dynamic ways ^8^. A complex systems approach requires bringing together all stakeholders from across the system to understand how, when and where to intervene. This means taking account of the key concepts of complex adaptive systems - emergence, feedback loops and adaptation, and using systems-focused interventions which may be partner-led, natural experiments or simulation studies that make use of data whilst also identifying what information is needed to support replicability ^9^. It also means using more credible methods of economic evaluation to inform social policy decisions, based on careful modelling of interacting social, fiscal and health outcomes and accounting for budget constraints and opportunity costs for different social groups. Across Europe, there are just a few examples of systems initiatives that have achieved reductions in child obesity prevalence and inequalities ^10, 11^, or that have delivered city-wide system change in ecology, technology, mobility and urban design to improve equity in the social determinants of health ^12^. UK transdisciplinary research is now needed that can change broader environments to improve the lives of our most vulnerable communities ^5^, focusing on children to yield high returns across the entire lifecourse ^1^.

In 2017, the UK Prevention Research Partnership (UKPRP), launched a novel model of public health funding to support research into the primary prevention of NCDs that could develop innovative and interdisciplinary approaches, and deliver upstream interventions to improve population health and reduce unfair health inequalities. We have been awarded 5-year UKPRP consortium funding to develop ActEarly City Collaboratories. “ *Cities are an ‘immense laboratory of trial and error, failure and success*” ^13^ and our city approach will provide real world opportunities to scope, deliver and evaluate sustainable and replicable population prevention interventions.

## Aims and objectives

Our long-term vision is to promote a healthier, fairer future for children living in deprived areas through a focus on improving environments that influence health and life chances.

Our objectives are:

1) To establish a prevention research consortium that unites broad transdisciplinary expertise including economics, geography, urban design, transport, education, housing, arts and culture, social justice and welfare (alongside the more usual public health sciences), with the public, policy leaders and practitioners from across our populations to develop shared understanding and priorities.

2) To identify, co-produce and implement system-wide early life upstream prevention solutions.

3) To provide efficient data platforms and methodological expertise enabling robust population-scale evaluation of the impact of interventions on environments, health related behaviours and interlinked health, educational, social and economic outcomes.

4) To evaluate, refine, replicate and disseminate our City Collaboratory approach as a model for addressing upstream determinants of health and inequality.

## Development of City Collaboratories

City Collaboratories in areas of high child poverty will provide research-ready, people-powered and data-linked test beds to co-produce, implement and evaluate multiple novel early life interventions to prevent disease and reduce inequalities. Our City Collaboratory approach will provide a whole-system environment where the public, scientists, policy leaders and practitioners work with each other to develop and test system-wide early life upstream prevention solutions, supported by efficient platforms for robust evaluation. We will create City Collaboratories first in Bradford (a post-industrial city in the North of England), and then in Tower Hamlets (a London borough) to support the identification, implementation and evaluation of upstream interventions in areas with high levels of child poverty. Our initial focus will be in Bradford, which provides a research-ready population laboratory for prevention research due to: its level of need, research track record, strong data linkage, pipeline of interventions and deep engagement with the community and local policymakers. Bradford, the fifth-largest city in the UK, has high levels of poverty and ill-health. It is ethnically diverse, with a large South Asian community and an accelerating prevalence of diabetes and cardiovascular disease ^14^. Researchers working with policymakers have built strong networks across health care providers and schools, connecting multiple systems and developing whole-system information and analytic capacity. We have worked closely with our communities to promote a strong public voice in the focus and delivery of research and now have a population based, system wide research infrastructure with committed investment to support the delivery of interdisciplinary preventative interventions.

Tower Hamlets, the London ActEarly Collaboratory site, will help explore replicability of the model and generalisability of interventions. Similar to Bradford, this East London borough is ethnically diverse and has some of the highest rates of child poverty in the UK, but it also has a strong foundation of community-developed research, in particular through the Communities Driving Change programme and transformative community health models developed by the Bromley-by-Bow Centre. The local authority has demonstrated enthusiasm and commitment for ActEarly and offers an existing platform of linked routine data through the Whole Systems Data Integration programme.

**Figure 1.**
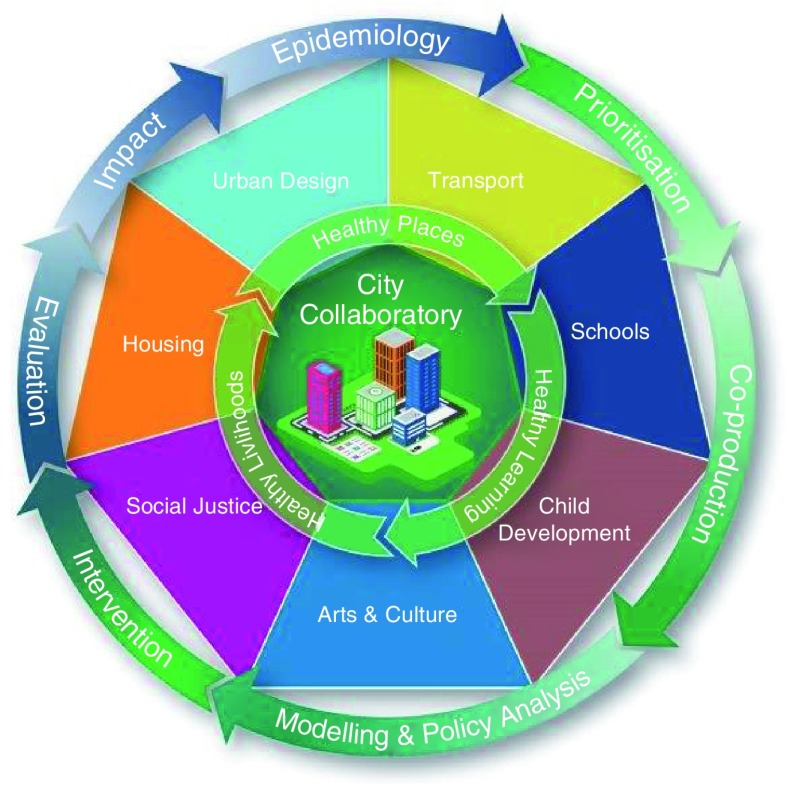
The City Collaboratory model utilises wide interdisciplinary expertise based in three inter-linked themes: Healthy Places, Healthy Learning and Healthy Livelihoods.

The Collaboratory model consists of a multistep interactive cycle that places local communities at the heart of decision making and active participation in both shaping and using the research, and connects academic expertise with real-world policymakers (see Figure 1). This cycle consists of: a) raising ideas (informed by evidence synthesis of epidemiological and other sciences), b) moving them through a critical cycle of engagement with stakeholders and by using Citizen Sciences ^15^, c) co-producing prioritised intervention strategies using internal and external experts, d) implementation (using whatever method is deemed optimal) and e) evaluating impact. Our evaluation will explore process (of the Collaboratory model itself and of interventions) and outcome (health and wellbeing, inequality outcomes, associated costs).

We will apply our ActEarly Collaboratory model to work programmes of three inter-linked themes that were identified collectively during the development phase of our programme: Healthy Places, Healthy Learning and Healthy Livelihoods ( Figure 1). These themes are underpinned by an emphasis on co-production and informed by our logic model (see below).

## Co-production of evidence with users

We are committed to genuine co-production with users in order to achieve acceptable, feasible, replicable, and sustainable systems interventions with real impact. We will engage policymakers, third sector organisations and our public (especially young people and their families) using citizen science methods of community engagement and prioritization in an asset-based community development approach ^16^ to sustainable community-driven change, building on work already started. Evidence shows that interventions engaging community members in delivery are effective, but no particular model of engagement appears superior ^17^, so we will test multiple approaches to support co-production ranging from robust consultation and dialogue, to complete multidisciplinary community and stakeholder led co-production. 

We have started our co-production activities, developing, refining and prioritizing the suite of activities outlined in each work programme with all key user groups. We have already identified synergies between community needs, policy and decision maker, and other user group priorities across our programmes and sites, for example: safer streets (places), healthy vending options in school (learning), easier access to welfare advice (livelihoods). Within each theme, we will start by eliciting user insights and experiences around prioritised topics using a tool kit of citizen science approaches (e.g. participatory mapping ^18^, extreme citizen science) ^19^ and traditional methods for consultation, dialogue and priority setting (e.g. open space). We will then refine and adapt existing co-production methods (e.g. experience-based co-design) ^20^ to unite communities, policymakers and researchers in focused co-production groups. We will evaluate the impact and effectiveness of our co-production activities on processes, outputs and impacts using mixed qualitative and quantitative methods. This will help develop and deliver appropriate engagement models across our sites and inform implementation of co-production activities within ActEarly.

## Logic model

**Figure 2.**
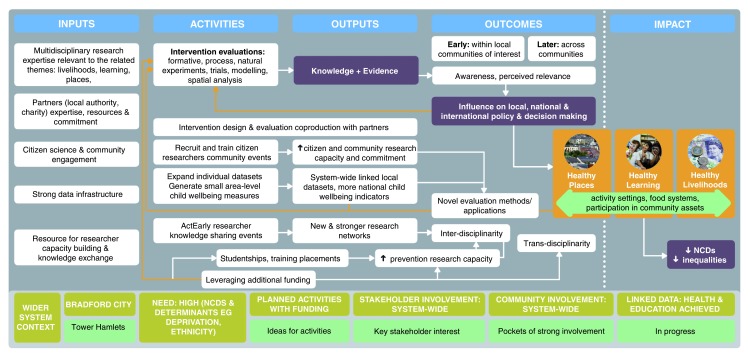
ActEarly logic model.

ActEarly’s logic model ( Figure 2) builds on a supportive system context (model base, green) of high need for ActEarly in both areas and capacity to intervene, which is particularly advanced in Bradford. Our key *inputs* include ActEarly’s wide multidisciplinary research team and strong partner support; its defining strengths, citizen science/co-production and rich data infrastructure, will be further enhanced. Our key *outputs* will be knowledge and evidence. Capacity building for current and potential researchers supports all ActEarly *activities* by promoting researcher skills, career entry and progression and leveraging further funding to support more evaluation (yellow arrow). *Outcomes* of novel methods in turn will increase the scale and scope of evaluation (yellow arrow). Promoting awareness of our outputs will enable ActEarly to influence decision making. Decision makers’ responses will also influence intervention development & evaluations (yellow arrow). The underpinning data infrastructures enables examination of longer-term *impacts*.

## ActEarly themes

### Healthy places

The spaces we live in affect how we travel, exercise, eat, socialise and interact. Deprived areas are often unhealthy obesogenic environments for children and young people, with physical infrastructure that increases barriers to healthy living such as physical activity (busy roads, lack of street connectivity, poor-quality green spaces ^21^), and healthy eating (fast food outlets ^22^). These are compounded by factors such as fear of crime and hostile traffic ^23^, lack of well-connected routes to work and leisure activities ^24^, low levels of social cohesion ^25^ and unfit and overcrowded housing. Research shows links between poorly designed neighbourhoods and obesity, mental health and cardiovascular health ^26– 29^, as well as poor social connectedness with community assets (arts, culture, parks, libraries, leisure centres, volunteer associations, social and community groups). Social networks and social capital are weakest in the most deprived areas ^30^. Thus, modifying local ‘places’ to make it easier for disadvantaged families to live better lives may offer gains across a range of outcomes.

We aim to increase the health and social potential of local places for children and young people by both changing physical infrastructure and by promoting connected communities. Improvements to physical environment will be based on the ‘healthy streets’ approach ^31^, which outlines indicators to promote healthy places: Everyone feels welcome, People to choose to walk and cycle, People feel relaxed, Easy to cross, Clean air, Not too noisy, Places to stop and rest, People feel safe, Things to see and do. Better connections within and across communities will be developed through locally led interventions including Bradford Metropolitan District Council’s (BMDC’s) Living Well programme which includes a healthy charter for businesses and communities, and a Tower Hamlets-based Community Engagement Campaign programme to encourage use of social, cultural and community assets. We will also evaluate natural experiments, for example Bradford’s Clean Air Zone, which is due to be implemented in 2020. All these interventions should impact on key lifelong health outcomes including prevalence of obesity, mental wellbeing and social outcomes, such as opportunity and social mobility. In the short term, we expect impact at an individual level (increased physical activity, active school travel; reduced exposure to emissions, improved eating behaviours), at the community level (increased use of open spaces; better social connectedness and more community participation) and at an environmental level (community assets and social infrastructure e.g. more safe play areas, better air quality, improved food environment). From a systems perspective, changing the attributes of place will change how communities move and thrive in that place thus creating interconnections between the ActEarly themes and delivering change across the whole system.

Some examples of our Healthy Places research questions include the following: Can ActEarly Healthy Places interventions:

1) Reduce child obesity and improve child mental wellbeing?
2) Increase active travel?
3) Increase the use and quality of open spaces for play and recreation?
4) Increase social connectedness?
5) Improve air quality?
6) Reduce health inequalities?
7) Increase uptake of housing related services (rights, advice, improvements, repairs) and maximise benefits of new housing?

### Healthy learning

Literacy and educational status are major upstream determinants of health, but educational opportunities are often poor for those living in disadvantaged areas ^32^, maintaining intergenerational cycles of inequality ^33^. Learning environments can play a critical role in encouraging children to adopt healthy lifestyles and nurturing the social and emotional wellbeing critical to good health ^34^. Learning is linked to place, and can provide powerful settings for community interactions which can support good parenting (e.g. parental engagement networks), and opportunities for wider health and social care (e.g. access to services and welfare advice). Previous attempts to introduce health programmes to schools (e.g. Social and Emotional Aspects of Learning (SEAL), the National Healthy Schools programme) have often produced minimal change ^35^. A new approach is needed that can move beyond a collection of individual health-promoting activities and deliver a real step change. Bradford is a UK Department for Education Opportunity Area with a delivery plan to improve opportunities for children and young people in the city. Via this initiative, we have established an ‘evidence active network’ (EAN) involving all learning environments across Bradford. The EAN will enable schools (n = 206) and pre-schools to adopt evidence-based practice in teaching and learning and empower staff and children to participate in research as citizen scientists. ActEarly offers an opportunity to widen the EAN’s remit to include health and wellbeing, and develop and deliver large scale evaluations - first in Bradford, with later extension and evaluation in Tower Hamlets. We will work with Evidence Champions on schools’ senior management boards to co-produce, deliver and evaluate health initiatives within local trailblazing sites, share learning across the network and develop an intervention ‘menu’. We will engage staff and students to create profiles of their environments as a baseline for monitoring progress against key indicators, such as absenteeism, attainment, child and adolescent mental health (CAMHS) referrals, exclusion, primary and secondary healthcare episodes.

Some examples of our Healthy Learning research questions include the following:

1) Does the EAN increase uptake of evidence-based interventions within early years and school settings?
2) Does the EAN increase participation of children, families, teachers, professionals and the community in research?
3) Which interventions have the greatest impact on physical and mental health, social wellbeing and educational attainment?

### Healthy livelihoods

People’s livelihoods are important for both their mental and physical health, partly due to higher levels of education, income and social class providing material benefits for health at all ages, but also through psychosocial mechanisms – dignity, sense (and locus) of control and self-worth, engagement in meaningful activity, social networks/social capital, social status ^36^. Demands are higher and resources lower at two critical stages: during the transition to parenthood and in early childhood ^37^, and during young people’s transitions from school into adulthood ^38^. Our aim is to develop and evaluate interventions and initiatives to address child, young person and family wellbeing and opportunities through increasing income, skills and control over community resources. The interventions will be co-produced with communities and include promoting take up of existing policy measures (2-year-old early education offer, co-locating welfare benefits in maternity services), pioneering interventions (universal basic income (UBI) and skills) and scaling-up promising interventions in new contexts (participatory budgeting). Outcomes we anticipate being affected in the short term are both child/adolescent/young adult/family-centred and place-based: i) child (social skills and cognitive abilities age 3; participation and enjoyment, school readiness); ii) young person (employment, training, education, participation, social and cultural capital, mental health and wellbeing); iii) family (maternal employment, family stress, paternal involvement, maternal health and wellbeing); iv) community (cohesion, neighbourliness), all of which affect the risk of NCDs.

Some examples of our Healthy Livelihoods research questions include the following:

1) What are some of the obstacles to the uptake of early childhood education and care, what interventions might increase appropriate uptake and what is their effect on key outcomes?
2) Can a UBI for young adults improve self-efficacy, mental health & wellbeing and engagement with education, employment, training and entrepreneurship? Is this more or less or cost-effective or effective when combined with life skills training?
3) Does community involvement (e.g. in participatory budgeting, a poverty commission or a social inclusion currency) improve individual self-efficacy, social support and health, and community wellbeing?

Examples of early interventions identified in our three themes are described in Table 1. We will develop logic models or causal loop diagrams for the design of interventions and their evaluation, and will promote strong cross-theme collaboration, for example where interventions across themes share common aims, populations or outcomes.

**Table 1.** Proposed ActEarly interventions in years 1–2.

Healthy Places			
Research question examples	Intervention examples	Outcomes	Funding
Can ActEarly Healthy Places interventions: 1) Reduce child obesity & improve child mental wellbeing? 2) Increase active travel to school? 3) Increase the use & quality of open spaces for play & recreation? 4) Increase social connectedness? 5) Improve air quality? 6) Reduce health inequalities? 7) Increase uptake of housing services and maximize benefits of new housing?	RESEARCHER LED: Combinations of diverse urban environment investments (Healthy Streets indicators) NATURAL EXPERIMENTS: Planning restrictions on fast food outlets near schools; Pricing & improving quality of vending foods/drinks; Soft drinks levy SIMULATION STUDIES: Simulation models of air quality to identify factors associated with poor air quality, estimate health effects & support improvement strategies	*Short term*: consumption of sugar sweetened drinks; density of fast food outlets; public transport use; physical activity levels *Medium term*: BMI in children & adolescents; air quality; urban environment improvements *Long term*: NCD prevalence; health inequalities	Planning restrictions already being implemented through BDMC Hot Food Takeaways Supplementary Planning Document (2014); Vending quality & pricing funded through leverage with industry partners; soft drinks levy introduced by central government; Healthy Streets funded from multiple sources (e.g. Active Bradford; BDMC; Sustrans)
Healthy Learning			
Research question examples	Intervention examples	Outcomes	Funding
1) Does the EAN increase uptake of evidence based health interventions within early years and school settings? 2) Which interventions have the greatest impact on physical and mental health, social wellbeing and education attainment?	RESEARCHER LED: Learning settings as community/advice venues; Standing desk; Glasses for Classes NATURAL EXPERIMENTS: Extension of ‘50 things to do before you’re 5’; BMDC Living Well charter, Free school meals SIMULATION STUDIES: Simulation models of more costly and radical variants of these investments in urban and school food environments	*Short term*: Community use of learning buildings; Number accessing advice; Number downloading 50 things app; Schools enrolled in Living Well; Uptake of school meals *Medium term*: BMI in children & adolescents; social connectedness *Long term*: NCD prevalence; health inequalities	Venues funded by BMDC & local businesses; Standing desks funded by Active Bradford; Glasses for Classes externally funded; Living Well funded by BMDC
Healthy Livelihoods			
Research question examples	Intervention examples	Outcomes	Funding
1) Can a UBI for young adults improve self-efficacy, mental health & engagement with education, employment & training? 2) Does community involvement (participatory budgeting) improve individual self- efficacy, social support and health?	RESEARCHER LED: Participatory budgeting to bring people together around specific projects in their community NATURAL EXPERIMENTS: Impact of universal credit on family income SIMULATION STUDIES: Simulation models of the impact of UBI on mental health, training, employment, crime	*Short term*: community participation; number of young people in training *Medium term*: children living in poverty; unemployment; education & training attainment *Long term*: NCD prevalence; health inequalities	Participatory budgeting: local budgets are allocated and spent by the community Universal basic income funded by taxation

## Evaluation framework

Complex systems theory and complexity thinking provide the framework for our analysis of systems focusing on the relational nature of systems, and the resulting emergent properties of those relations. ActEarly aims to evaluate the impacts of interventions in a complex system setting on the health of children in Bradford and Tower Hamlets across the three themes. We will collect and link bespoke and available data at individual and area-level to enable evaluation of multiple initiatives.

### Outcomes

For all interventions, combinations of interventions, and systems we will be interested in (a) understanding processes of implementation including adaptation, (b) effectiveness and (c) economic impact. We will estimate both average and distributional effects, harnessing our consented cohort and routine linked datasets to follow up our children’s longer-term health and wellbeing outcomes.

### Single intervention evaluations

We will use a variety of methods (see Table 2), depending on context, with a preference for quasi-experimental methods (e.g. propensity score matching, regression discontinuity designs, difference-in-differences) where possible. Qualitative data collection will give insights into how interventions achieved (or not) their effects, paying particular attention to contextual factors, and to identify any unexpected impacts.

**Table 2.** Evaluative principles, methods and approaches.

*Evaluative principles, methods and approaches*	
Ground evaluations in theories of change	Logic models and theories of change will shape evaluation. We will seek a shared understanding of expected intervention mechanisms, salient outcomes (intended and unintended) and information needed for decision makers, informed by relevant mid-range sociological and psychological theories
Conduct implementation evaluations	Timely investigation and feedback will shape and adapt intervention development and delivery
Maximise use of experiments, natural experiments and quasi-experimental designs	We will maximize evaluability ^37^ through influencing intervention introduction, enabling use of trials within cohorts, e.g. RCT of UBI (Healthy Livelihoods), cluster RCTs of school based interventions (Healthy Learning), controlled before-and-after designs to evaluate green space improvements (Healthy Places). Where full multicentre trials are merited, we will leverage additional funding with our Clinical Trials Unit partners (Bryant). Where we cannot influence intervention rollout, we will use natural experiment/ quasi-experimental designs in line with MRC guidance (e.g. difference-in- difference, interrupted time series, propensity score matching, triple differences, synthetic controls, instrumental variables, RDD)
Study process as well as outcomes	We will include measurement of acceptability, adoption, appropriateness, cost, feasibility, fidelity, penetration and sustainability informed by relevant theoretical frameworks e.g. RE-AIM and/or Consolidated Framework for Implementation Research. Qualitative approaches and quantitative methods e.g. causal mediation analysis will investigate potential mechanisms of action
Capture distributional effects	Evaluations will be designed to capture effects on inequalities (e.g. by ethnicity and deprivation) as well as overall changes in outcomes
Use ActEarly Data Platforms	Embedding evaluations in ActEarly Data Platforms will enable population scale studies and substantially reducing the measurement burden.
Citizen Science	We will supplement existing data through citizen science data collection approaches
Qualitative methods	We will use established (interviews, focus groups, documentary analysis, ethnography) and develop novel (e.g. child-centred, visual) qualitative data collection methods
Economic metrics	Tailored for decision makers (e.g. cost-utility, cost-effectiveness, cost-benefit, return on investment, budget impact). Costs and benefits falling on different parts of the system e.g. health, education, local authorities

### Evaluating combinations of interventions

We will treat ‘groups of conditions’, (combinations of interventions that may interact to produce effects through ‘conjunctural causation’) as *cases* and use qualitative comparative analysis (QCA) ^39^ as our approach. This algebraic technique will be used to test the extent to which different components of the configuration of the interventions, and their context, seem necessary or sufficient to produce outcomes. It will allow us to examine pathways to both positive and negative (unintended) outcomes across our complex system. Definition of cases can be based on both quantitative and qualitative data, the configurations of which are analysed as either ‘crisp set’ (where data are clearly binary) or ‘fuzzy set’ (allowing for calibration to a scale where they are not). 

### Whole, complex system analyses

We will co-develop complex systems maps ^40^, and/or complex networks of systems, related to each of our research themes or related to producing outcomes in, for example, early childhood, school age children or young adults, or systems related to particular stakeholder spheres of influence, e.g., local authorities. Systems will be developed and described through iterative concept mapping, expert input (via Delphi surveys) and then simulated (and refined) via agent-based and/or system dynamic models. Empirical data will then be used to explore the credibility of, and to improve, the system models. 

### Life course analysis, policy and economic modelling

We will simulate long-term policy effects, public costs and inequality impacts of interventions, sets of interventions or systems for our Collaboratory sites and nationally, building on microsimulation methods ^41^. Simulation methods will explore long-term impact of interventions on health using relationships between early life predictors and later disease.

### Meta-evaluation of the ActEarly Collaboratory

We will adopt a realist context-mechanism -outcome perspective. This will involve multiple components including QCA, a complex systems analysis of the whole Collaboratory, social network analysis of collaborators, qualitative interviews, and tracking of processes, outputs and impact on policymaking.

## Data tapestry

Our evaluation plans will require efficient use of our bespoke and routine data sources. We benefit from well-established data platforms in both Bradford and Tower Hamlets. Bradford has some of the most richly described populations in the UK with its cohorts and Connected Bradford dataset which includes linked health, social care and education routine data for 700,000 citizens. Tower Hamlets is establishing a parallel Whole System Demonstrator-linked dataset, and both settings have well characterized geospatial data on exposures such as pollution, green space, transport, connectivity, walkability and fast food outlets.

**Figure 3.**
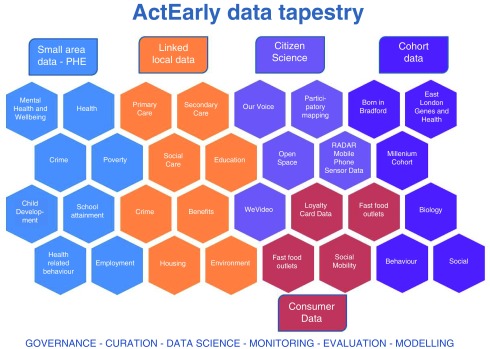
ActEarly data tapestry.

Our goal is to develop safe and secure data tapestries that unite our cohort, routine data, public health data, consumer data and citizen science, and allow us to evaluate multiple, interdependent outcomes across the life course ( Figure 3).

## ActEarly knowledge transfer and exchange (KTE)

We will develop effective KTE activities to promote the uptake of actionable evidence. At the core of our KTE strategy will be the building of trusted and enduring partnerships between researchers, practitioners and policymakers, promoting effective dissemination and communication developed from the best implementation science and drawing on professional communications and marketing expertise (see communication plan). ActEarly will favour an integrated approach that incorporates direct audience engagement with information push, pull, linkage and exchange. The development of specific knowledge mobilisation activities for the consortium’s proposed research outputs will be theory driven and informed by the topic, research findings, and the needs and preferences of the audiences to be targeted. Our goal is to produce evidence that is useful to decision makers.

ActEarly partners involved in our KTE include the What Works Network, Centre for Cities, Local Government Association, NESTA, Academy of Urbanism, Sustrans, Royal Society for Arts, Public Health England, National Housing Association and the UK National Institute for Health Research.

## Challenges

This is an ambitious research programme that aims to catalyse system-wide transformation underpinned by strong co-production and robust evaluation of multiple and interacting health promoting interventions across two city regions. We face a number of challenges in implementing such a programme. Effective community engagement will be central to the prioritisation and uptake of interventions. Key to this success is being sensitive to the complexity of the setting and understanding the need to reconcile differing agendas. We will use multi-faceted approaches that capitalise on traditional strengths and assets but also develops novel methods of citizen science and participatory research.

We will need to embed our research within mainstream policy and practice and work hard to achieve and maintain meaningful engagement between communities, researchers, local authorities and local and national stakeholders in an era of severely constrained resources. We will be required to prioritise our activities to maximise value of limited resources and attract funding to support an ambitious pipeline of interventions. The curation and development of multiple combined data sources will need to be safe and secure but also accessible and more useful for prevention policy than the conventional randomised trial approach to causal inference. We also face methodological challenges in evaluating multiple and interacting population level interventions and natural experiments. Our meta-evaluation will capture and monitor our progress in tackling these challenges.

## Data availability

No data are associated with this article.
