# Supplementary material for: ActEarly: a City Collaboratory approach to early promotion of good health and wellbeing
Source: Wellcome Open Res. 2019 Oct 14;4:156. [Version 1] doi: 10.12688/wellcomeopenres.15443.1 (PMC6904987; doi:10.12688/wellcomeopenres.15443.1)
Supplement: Peer review history

# Peer review: Reviewer response for version 1

**DOI:** 10.21956/wellcomeopenres.16889.r37072

**Published:** 2019-12-10

**Authors:** Elly Howse, Lucie Rychetnik

**Affiliations:** 1 The Australian Prevention Partnership Centre, Sax Institute, Sydney, Australia; 2 School of Public Health, Faculty of Medicine and Health, The University of Sydney, Sydney, Australia; 3 Prevention Research Collaboration, School of Public Health, Faculty of Medicine and Health, The University of Sydney, Sydney, Australia

This Open Letter is an important take on a challenging area for chronic disease prevention - namely the ‘how’ of putting prevention research into practice, instead of simply describing the ‘what’ (the problem of chronic disease). This paper sets out an impressive program of work and research which is ambitious and comprehensive. The researchers should be commended for this and for engaging with systems thinking in an applied way.

 The background, aims and objectives are clearly described, as are the major themes for the project. The challenge for this paper was in outlining and explaining the overall elements of the project with sufficient detail in a way that does not feel like there are lots of bits and pieces. This is where Table 1 on page 9 with intervention examples is particularly helpful, and the data tapestry (Figure 3) is a useful way of identifying all the different data components of the project. However, the evaluation components are included in a separate table, which does make it a little difficult to link the evaluation framework with the broader project.

 The authors have developed a logic model to support the program, which is understandable given others such as Foster-Fishman and Watson ^1^ have argued a logic model (or a ‘theory of change’) is an essential part of a systems approach. However it was less clear how this rather linear logic model fit within the authors’ definition of a ‘supportive system context’ in terms of the program.

 The authors briefly explain in the ‘Evaluation framework’ section why complex systems theory and complexity thinking are being used, but it is worthwhile to reflect on complex systems approaches to evaluation, which consider feasibility and appropriateness of complex program evaluations within real world settings ^2^. The researchers have demonstrated how complex systems thinking is informing the intervention but less so for the evaluation framework. This would address the point raised earlier about clearly linking the different evaluation components with the intervention components.

 In the section on co-production, it is necessary to define co-production beyond describing the importance of engaging and empowering citizens through citizen science or participatory action research. For example, given this is an initiative bringing together researchers and policymakers, it is helpful to reflect on why co-production between these two groups is needed, particularly in terms of prevention, or what the existing evidence base is for co-production for prevention ^3^^,^^4^ and how this impressive program of work and evaluation will add to it.

We confirm that we have read this submission and believe that we have an appropriate level of expertise to confirm that it is of an acceptable scientific standard.

## References

[1] : The ABLe change framework: a conceptual and methodological tool for promoting systems change. *Am J Community Psychol*.2012;49(3-4) : 10.1007/s10464-011-9454-x 503-16 10.1007/s10464-011-9454-x 21735334

[2] : Applying pragmatic approaches to complex program evaluation: A case study of implementation of the New South Wales Get Healthy at Work program. *Health Promot J Austr*.2019;30(3) : 10.1002/hpja.239 422-432 10.1002/hpja.239 30860630

[3] : Partnering to prevent chronic disease: reflections and achievements from The Australian Prevention Partnership Centre. *Public Health Res Pract*.2018;28(3) : 10.17061/phrp2831821 10.17061/phrp2831821 30406262

[4] : Knowledge mobilisation for chronic disease prevention: the case of the Australian Prevention Partnership Centre. *Health Res Policy Syst*.2018;16(1) : 10.1186/s12961-018-0379-9 109 10.1186/s12961-018-0379-9 30445963PMC6240292

# Peer review: Reviewer response for version 1

**DOI:** 10.21956/wellcomeopenres.16889.r36755

**Published:** 2019-10-18

**Authors:** Petra S. Meier

**Affiliations:** 1 School of Health and Related Research, University of Sheffield, Sheffield, UK

The open letter describes a large and ambitious consortium research project commissioned under the UK Prevention Research Programme. In a similar vein to trial pre-registrations, this letter sets out to pre-register the team's plans to set up two City Collaboratories focused for whole-systems research on influences in childhood and adolescence that shape health outcomes over the lifecourse. 

 This is clearly a very well thought out project, and it has prevailed through several stages of rigorous selection and peer review, so there is not really anything left for me to critique in terms of scientific quality. The authors present a strong, multi-pronged evaluation framework that allows them to reflect on whether ActEarly is achieving its milestones.

I confirm that I have read this submission and believe that I have an appropriate level of expertise to confirm that it is of an acceptable scientific standard.
